# The Role of High Mobility Group Box 1 in Ischemic Stroke

**DOI:** 10.3389/fncel.2019.00127

**Published:** 2019-04-02

**Authors:** Yingze Ye, Zhi Zeng, Tong Jin, Hongfei Zhang, Xiaoxing Xiong, Lijuan Gu

**Affiliations:** ^1^Central Laboratory, Renmin Hospital of Wuhan University, Wuhan, China; ^2^Department of Pathology, Renmin Hospital of Wuhan University, Wuhan, China; ^3^Department of Neurosurgery, Renmin Hospital of Wuhan University, Wuhan, China; ^4^Department of Anesthesiology, Zhujiang Hospital of Southern Medical University, Guangzhou, China

**Keywords:** ischemic stroke, high-mobility group box 1 protein (HMGB1), inflammatory response, stroke-induced immunodepression, signaling pathways

## Abstract

High-mobility group box 1 protein (HMGB1) is a novel, cytokine-like, and ubiquitous, highly conserved, nuclear protein that can be actively secreted by microglia or passively released by necrotic neurons. Ischemic stroke is a leading cause of death and disability worldwide, and the outcome is dependent on the amount of hypoxia-related neuronal death in the cerebral ischemic region. Acting as an endogenous danger-associated molecular pattern (DAMP) protein, HMGB1 mediates cerebral inflammation and brain injury and participates in the pathogenesis of ischemic stroke. It is thought that HMGB1 signals *via* its presumed receptors, such as toll-like receptors (TLRs), matrix metalloproteinase (MMP) enzymes, and receptor for advanced glycation end products (RAGEs) during ischemic stroke. In addition, the release of HMGB1 from the brain into the bloodstream influences peripheral immune cells. However, the role of HMGB1 in ischemic stroke may be more complex than this and has not yet been clarified. Here, we summarize and review the research into HMGB1 in ischemic stroke.

## Introduction

Ischemic stroke, caused by the formation of a clot in a main cerebral blood vessel, results in a sharp drop in perfusion of the ischemic core and is a leading cause of death and disability worldwide. An ischemic stroke deprives the brain of oxygen and nutrients, leading to permanent necrotic neuronal death in the region of brain tissue supplied by the affected cerebral artery (Singh et al., 2016). The pathophysiology of the complex brain injury that occurs following an ischemic stroke has not yet been fully elucidated. Cerebral ischemia–reperfusion causes a loss of cellular ion homeostasis, activation of caspases, generation of reactive oxygen species (ROS), bioenergetic failure, impaired mitochondrial function, and excitotoxicity in brain tissue, but these complex reactions are not yet fully understood. Activation of complementary pathways promotes the generation of arachidonic acid products and cytokines, infiltration of immune cells, and disruption of the blood–brain barrier (BBB), which initiates inflammatory cascades (Fann et al., 2013; Ballarin and Tymianski, 2018; Bao et al., 2018).

Stroke-induced inflammation and the activation of proinflammatory mediators have been the focus of recent research into the mechanisms of stroke-induced brain damage (Chen et al., 2017; Stonesifer et al., 2017), and high-mobility group box 1 protein (HMGB1), a typical damage-associated protein, has gained particular interest (Choi et al., 2018; Shichita et al., 2017). The HMGB proteins were first identified by Goodwin et al. (1973). Since then, a number of studies have confirmed that HMGB1 plays a central role in the pathogenesis of many diseases, including systemic lupus erythematosus (SLE), acute liver failure (ALF), tumors, and cerebrovascular diseases (Cully, 2013; Majumdar et al., 2013; Jian et al., 2016; Xiong et al., 2016; Seidu et al., 2017; Hossain et al., 2018). The HMGB1 protein was originally reported to be a ubiquitous, non-histone chromosomal protein that plays a role in DNA replication and repair in eukaryotic cells (Liu et al., 2010; Thomas and Stott, 2012). When released or secreted, HMGB1 acts as a sentinel for the immune system and triggers cell survival or death pathways in response to stress or damage. It is a multifunctional protein, with the functions depending on its location in the cell. In normal brain tissue, HMGB1 is usually located in the nuclei. However, following a stroke, HMGB1 is translocated to the cytosol and secreted into the extracellular space. Studies have indicated that intracellular HMGB1 plays an important role in the regulation of energy homeostasis and transcription (Tang et al., 2016). In contrast, it has been reported that extracellular HMGB1 directs BBB breakdown, neuroimmune activities, and neuronal death (Gardella et al., 2002; Faraco et al., 2007).

In this review article, we aim to illustrate the biological functions of HMGB1 and the role of this protein in ischemic stroke, as well as fully clarify the mechanisms of HMGB1’s role in stroke pathology, to highlight HMGB1 and pathways which may be potential drug targets in an attempt to provide new prospects and directions for the treatment of ischemic stroke.

## Structure and Characteristics of HMGB1

The HMGB protein family comprises four proteins: HMGB1, HMGB2, HMGB3, and HMGB4. All have HMG box domains, which are DNA-binding motifs (Stros, 2010). HMGB1 protein is highly conserved in evolution as a chromatin-binding molecule, and since its discovery in 1973, it has attracted the attention of researchers (Goodwin et al., 1973).

**Figure 1:**
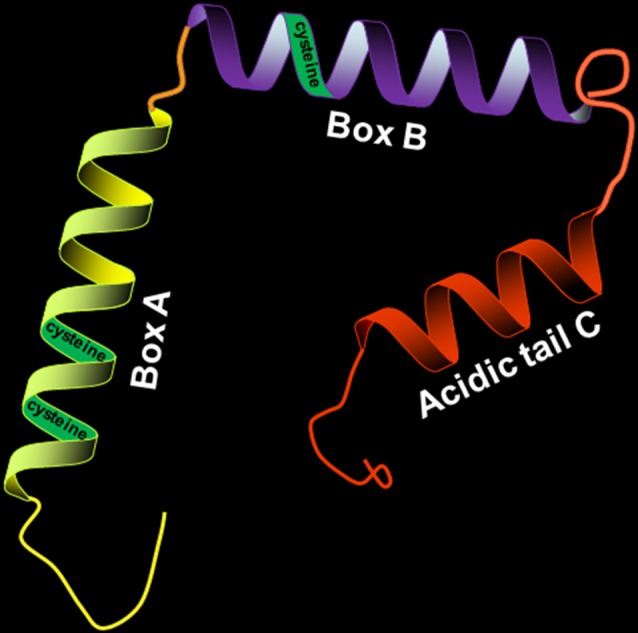
Structure of high-mobility group box 1 protein (HMGB1; 30 kD, 214 amino acids). HMGB1 is divided into three distinct structural domains: A box, B box, and C tail. The three regions have their respective positions. The three cysteines (C23, C45, and C106) in the molecular structure of HMGB1 contribute to its redox state.

HMGB1 has a molecular weight of 30 kD and consists of 214 amino acid residues. Its amino acid sequence is highly conserved, with over 98% homology between humans and rodents. HMGB1 consists of three distinct structural domains: two positively charged DNA-binding motifs (boxes A and B) and a highly negatively charged C-terminal acidic tail (Figure 1). The A box is located at the N-terminal, and the B box is between the A box and the C tail. The A and B boxes are evolutionarily conserved and are each composed of three α-helical structures, which can nonspecifically bind to DNA. The A box is important for anti-inflammatory action (Lotze and Tracey, 2005), while the B box is critical for proinflammatory activity and cell differentiation (Sparatore et al., 2001). However, the most important part of HMGB1 is the C-terminal domain, which is associated with regulating HMGB1’s DNA binding affinity.

There are three cysteines (C23, C45, and C106) in the molecular structure of HMGB1 (Yang et al., 2013), and the redox state of these three cysteines regulates biological activity and receptor binding. HMGB1 with all-thiol state of the three cysteine residues has been reported to cooperate with C-X-C motif chemokine 12 (CXCL12) to form a heterocomplex with chemotactic activity, which binds to the CXCL12 reciprocal receptor C-X-C chemokine receptor type 4 (CXCR4) in a synergistic fashion, contributing to the cytokine-inducing and chemoattractant activities of HMGB1 (Schiraldi et al., 2012; Venereau et al., 2012). However, it is reported that all-thiol HMGB1 is oxidized, leading to the formation of disulfide state at the three cysteine residues, when it is released into the circulation after cerebral ischemia. Oxidized HMGB1 possesses a cytokine-stimulating function, inducing the translocation nuclear factor “kappa-light-chain-enhancer” of activated B-cells (NF-κB) to the nucleus and synthesis of tumor necrosis factor alpha (TNF-α) in activated macrophages (Yang et al., 2012; Singh et al., 2016).

## The Biological Functions of HMGB1

Under physiological conditions, as a non-histone chromosome binding protein, HMGB1 remains in the nucleus and nonspecifically binds to DNA, stabilizes nucleosomes, and assists in DNA replication and transcription (Liu et al., 2010; Thomas and Stott, 2012; Tang et al., 2016). In addition, HMGB1 may also interact with nucleotides to repair related proteins, play a role in DNA repair, and identify damaged DNA fragments and remove them. During the maturation of T and B lymphocytes, HMGB1 can affect the recombination, differentiation, and development of V(D)J gene fragments. Cell activation, injury, or death caused by some pathological conditions, such as hypoxia, result in HMGB1 translocation from nucleus to cytoplasm or extracellular space, due to the separation of HMGB1 with damaged DNA. The translocation of HMGB1 between the nucleus and the cytoplasm is associated with the acetylation of lysine in nuclear localization sites (NLSs; Andersson et al., 2018).

**Figure 2:**
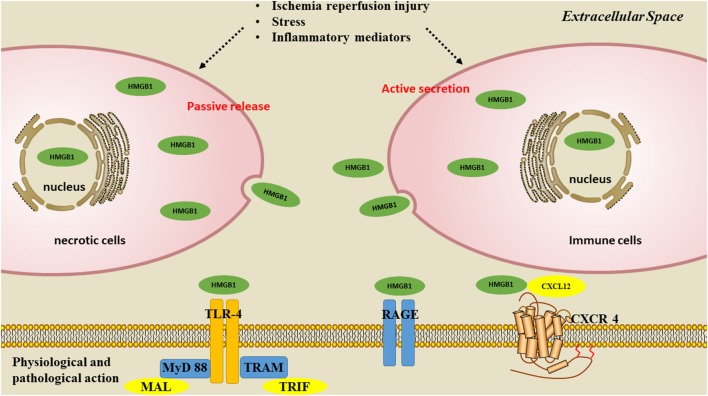
Pathways of HMGB1 secretion. There are two mechanisms used by cells to liberate HMGB1 into the extracellular milieu. Somatic cells contain large amounts of HMGB1 that is passively released into the extracellular space during cell apoptosis or necrosis. A second mechanism is the active secretion of HMGB1 from activated immune or nerve cells.

There are two ways for HMGB1 to be released: passive release or active secretion. During disease development, these two mechanisms are not completely independent but are mutually causal. It has been confirmed that the secretion of HMGB1 from necrotic cells functions as danger-associated molecular patterns (DAMPs), and contributes to the inflammatory cascade (Scaffidi et al., 2002; Singh et al., 2016). Okuma et al. (2012) reported that HMBG1 is secreted within a few hours of stroke onset and is a hyperacute DAMP that devastates the BBB. When HMGB1 is released into the extracellular space, it is recognized by receptors such as toll-like receptors 2 and 4 (TLR2, TLR4; Park et al., 2004); and receptor for advanced glycation end product (RAGE); (Rauvala and Rouhiainen, 2007), thereby activating the NF-κB signaling pathway and contributing to the inflammatory response (Lok et al., 2015) (Figure 2). Inhibiting the expression and translocation of HMGB1 and its receptors has been demonstrated to have anti-inflammatory and neuroprotective effects on ischemic injury (Tao et al., 2015).

## Receptors and Transduction Pathways of HMGB1

Although multiple receptors have been reported for HMGB1, only four receptors have been identified: TLR2, TLR4, RAGE, and CXCR4, as well as matrix metalloproteinases (MMPs; Hori et al., 1995; Carty and Bowie, 2011). Other receptors are likely to present molecules to bind HMGB1. The binding of HMGB1 to these receptors mediates inflammatory factors production and eventually results in systemic inflammation.

The transmembrane protein RAGE is a member of the immunoglobulin superfamily and is widely expressed in the cell surface of many cells including mononuclear macrophages, epithelial cells, endothelial cells, and nerve cells (Kokkola et al., 2005). It binds to a variety of active proteins, including glycated proteins, cytoplasmic protein S100, amyloid β-peptide, and HMGB1. Under physiological conditions, cells have low RAGE expression, but when its ligand molecules increase, its expression also increases. RAGE has a high affinity for HMGB1. When HMGB1 binds to the upregulated RAGE, some protein kinases in mitogen-activated protein kinase (MAPK) and phosphatidylinositol 3 kinase/protein kinase B (PI3K/Akt) pathways, such as p38 kinase, SAPK/JNK, extracellular regulated protein kinases1/2 (ERK 1/2) and Akt are activated by this mitogen (Qin et al., 2009; Tong et al., 2018); in addition, cell division cycle 42 (Cdc42), Ras-related C3 botulinum toxin substrate (Rac), and just another kinase/signal transducer and activator of transcription 1 (JAK/STAT1)-mediated signal transduction pathways are also activated (Tsoyi et al., 2010). These events finally lead to the translocation of NF-κB, inducing the expression of inflammatory cytokines and chemokines that participate in the maturation and migration of immune cells, the expression of surface receptors and growth of neurites, as well as tumor proliferation (Muhammad et al., 2008; Akirav et al., 2012; Zhang et al., 2017).

TLR2 and TLR4 are also HMGB1 receptors that induce a proinflammatory response (Yang et al., 2010, 2015). TLR-mediated signaling is important for cytokine release and activation of innate immunity. Kang and Lee demonstrated that HMGB1 binding to TLRs can mediate activation of certain pathways, including myeloid differentiation factor 88 (MyD88)-dependent and MyD88-independent pathways, which result in the expression of inflammatory genes, leading to the production of cytokines and chemokines (Kang and Lee, 2012). Recently, Kim E. J. et al. (2018) reported that binding of HMGB1 to TLR-4 increases interleukin-1β (IL-1β) production in vascular smooth muscle cells *via* the activation of Nod-like receptor protein 3 (NLRP3) inflammasome.

CXCR4 (a CXCL12 reciprocal receptor) is another receptor that is reported to bind HMGB1. A CXCL12/HMGB1 heterocomplex can interact with CXCR4 receptors and induce migration of inflammatory cells. Recently, Tirone et al. (2018) demonstrated that fully reduced HMGB1 (fr-HMGB1) orchestrates tissue regeneration *via* CXCR4.

## HMGB1 and Cerebral Ischemic Stroke

As the global population ages, cerebral ischemic stroke and its complications have become the main cause of disability and death worldwide (Neumann et al., 2015). During ischemic stroke, cerebral artery occlusion leads to oxygen and nutrient depletion in neural tissue (Lo, 2010). In response to ischemic injury, astrocytes and microglia in the brain activate and release reactive nitrogen species (RNS), ROS, and proinflammatory cytokines that cause secondary damage to the infarct area. Studies have shown that HMGB1 is involved in the pathogenesis of ischemic stroke and reperfusion injury (Kim et al., 2006, 2008; Liu et al., 2007; Muhammad et al., 2008; Qiu et al., 2008; Shichita et al., 2012). Furthermore, the application of anti-HMGB1 neutralizing antibodies has been shown to reduce infarct volume and ameliorate infarction after middle cerebral artery occlusion (MCAO) in rats (Kim et al., 2008). Recently, it has been reported that HMGB1 works as an immune system signal (or DAMP), and that HMGB1 inhibition has a protective effect against damage following an ischemic stroke. There are two phases of the post-ischemic stroke inflammatory response: the early phase, which is involved in neural tissue destruction, and the late phase, which consists of tissue remodeling (Agnello et al., 2002). In the early phase, HMGB1 is released from nerve cells to accelerate the inflammatory response. However, in the late phase, HMGB1 release from reactive astrocytes may affect the regeneration of nerves and blood vessels, and promote tissue remodeling (Bianchi et al., 2017).

### Process of Activation and Secretion of HMGB1

In the early stages of stroke, a large number of neurons undergo sustained hypoxia and oxidative toxicity. The cell membranes of neurons are destroyed, “holes” appear, and HMGB1, loosely bound to chromosomes, is passively released into the extracellular space (Tsung et al., 2007). Meanwhile, microglia and astrocytes are activated and intracellular HMGB1 is modified by a series of acetylation and phosphorylation reactions, thus decreasing its affinity for DNA, finally leading to active secretion of HMGB1 into extracellular space. This is accompanied by increases in RNS and ROS levels. These factors all contribute to the formation of highly oxidative conditions. The activity and function of many proinflammatory cytokines are adjusted by the oxidation of methionine, cysteine, and tyrosine residues (Singh et al., 2016). HMGB1 is also modified to the restored or oxidized forms, which have specific cellular functions in the ischemic brain (Zhang J. et al., 2011; Lorenzen et al., 2017). Once HMGB1 is released, an inflammation signal is emitted, and the immune system responds with a proportional positive feedback amplification.

In a model of MCAO in mice, Kim et al. (2008) reported that, through phosphorylation and acetylation, HMGB1 is translocated into the cytoplasm and then secreted into the extracellular area. Moreover, using *in vitro* experiments, Hua et al. (2007) demonstrated that when primary cultures of neurons undergo oxygen glucose deprivation (OGD), HMGB1 is detected in the liquid supernatant. Fully reduced HMGB1 has been shown to be prevalent in the serum and brain samples of mice after 2 h of stroke (Liesz et al., 2015). Furthermore, cytokine-inducing disulfide HMGB1 was discovered in mouse serum after 24 h of cerebral ischemia (Laird et al., 2014). These studies demonstrate that early necrotic brain tissue releases fully reduced HMGB1 to the blood stream, where it is oxidized to its cytokine-inducing form.

### Dynamics of HMGB1 in Ischemic Stroke

When an ischemic stroke occurs, HMGB1 is released into peripheral blood from the central nervous system (CNS). This extracellular HMGB1 has been demonstrated to provoke an inflammatory response in many experimental animal models (Yang et al., 2011). The HMGB1 levels in serum and plasma reflect the expression level of HMGB1 in the CNS and the extent of brain injury. In experimental animal models of cerebral ischemia–reperfusion injury (IRI) and in ischemic stroke patients, HMGB1 levels in the cerebral spinal fluid (CSF) and serum are significantly increased. Goldstein et al. (2006) reported that HMGB1 levels in ischemic stroke patients rapidly increase, and are up to 13 times higher than those in the control group within 24 h. Kim et al. (2008) reported that CSF and serum HMGB1 levels increase rapidly after 3 h of ischemic stroke, and generate 2 peaks: one on the 1st day and the other on the 6th and 7th days after stroke. The authors suggested that the HMGB1 peak on the 1st day may be caused by acute necrosis of nerve cells induced by excitotoxicity and that HMGB1 activates microglia in the early stages of inflammation. The second peak of HMGB1 after ischemic stroke, however, is thought to be secreted by various immune cells such as microglia, macrophages, astrocytes, and vascular endothelial cells. More recently, Umahara et al. (2018) also observed that, at the acute stage of cerebral infarction in patients, HMGB1 is located in the neuronal cytoplasm, while during the late stage of cerebral infarction, HMGB1 is mainly secreted by macrophages located in the basal ganglia and in some ischemic regions. Xiong et al. (2014, 2016) confirmed that HMGB1 transposes from the neuronal cell nucleus to the cytoplasm, and finally to the extracellular environment after ischemic stroke in the MCAO rats and mice. They found that HMGB1 is comprehensively expressed in the nuclei of neurons in the control group and significantly reduced after MCAO, and its subcellular translocation is observed at an early stage (12 h) of ischemic stroke (Xiong et al., 2014). At the same time, increased numbers of HMGB1 positive microglia/macrophages are observed infiltrating the stroke area and exacerbating inflammation (Xiong et al., 2014). It was suggested that at the early stage of stroke, HMGB1 is first passively released from the dying neurons, accompanied by active secretion by the actively infiltrated microglia/macrophages (Xiong et al., 2014). Meanwhile, they found that HMGB1 levels are increased in the CSF and circulation at 5, 24, and 48 h of reperfusion (Gu et al., 2013; Xiong et al., 2014, 2016). This phenomenon may be related to the disruption of the BBB and an increase of vascular permeability, or to other unknown causes. HMGB1 is reported to be highly expressed in the blood not only during the acute phase but also for at least 2 weeks following ischemia in rats (Kim et al., 2006), even longer to around 1 month in ischemic stroke patients (Schulze et al., 2013).

### Contribution of HMGB1 in Early Cerebral Ischemic Stroke

Early restoration of reperfusion is a fundamental step in the prevention of decreased brain perfusion in cerebral ischemia patients. However, while ischemia as a stimulus may activate macrophages to release the inflammatory mediator HMGB1, reperfusion may exacerbate the inflammatory response by stimulating the release of more HMGB1 into the extracellular environment and aggravating brain tissue damage. Kim et al. (2008) constructed an HMGB1 short hairpin RNA (shRNA) plasmid and injected it into MCAO mice to interfere with HMGB1 expression. This treatment reduces the size of cerebral infarcts in mice (Kim et al., 2008). Meanwhile, they also found that the activation and infiltration of microglia in the ischemic region is reduced with this treatment, and the expression of TNF-α, IL-1β, cyclo-oxygenase-2 (COX-2), and inducible nitric oxide synthase (iNOS) are decreased (Kim et al., 2008). The anti-HMGB1 antibody also significantly reduces the size of the cerebral infarct, improves the permeability of the BBB, inhibits the activation of microglia, and reduces the expression of TNF-α and iNOS in the MCAO mouse model (Liu et al., 2007; Muhammad et al., 2008). Conversely, the infarct volume is increased and the extent of inflammation response is aggravated when recombinant HMGB1 is injected into mice (Goldstein et al., 2006). After treatment with glycyrrhizin, a direct inhibitor of HMGB1, the level of HMGB1 in neuronal cells is significantly increased, while translocation and release of HMGB1 are inhibited, neuronal death in the infarct areas is significantly reduced, as is infarct volume. This was accompanied by a reduction in the activation and infiltration of inflammatory cells including microglia/macrophages, neutrophils and T lymphocytes, as well as the production of proinflammatory cytokine TNF-α, IL-1β and interferon-γ (IFN-γ; Kim et al., 2012; Gu et al., 2013; Xiong et al., 2016). HMGB1 is also expressed in microglia, and the extent of cerebral infarct can be significantly reduced by the inhibition of microglial HMGB1 expression (Hayakawa et al., 2013). Recently, Balosso et al. (2014) reported that different redox forms of HMGB1 can induce differential activation patterns of microglia and that disulfide HMGB1 may promote neuronal cell death induced by N-methyl-D-aspartic (NMDA) acid receptor through TLR-4 receptors.

Extracellular HMGB1 acts as a proinflammatory factor and activates microglia and macrophages to amplify inflammatory responses by recognizing TLRs or RAGE receptors. The RAGE receptor is involved in cerebral ischemic injury caused by HMGB1, and the level of soluble RAGE (sRAGE) in the serum of patients with acute stroke is significantly increased. The expression of RAGE is also increased in the brain tissue of patients with unilateral cerebral infarction. Liesz et al. (2015) reported that HMGB1 is released at the acute stage of ischemia from the injured brain in both the mice model and patients, and HMGB1-RAGE signaling participates in the ischemic stroke, which was believed to be critical for clarifying the mechanism of the brain–immune interaction after ischemia. The TLRs are also implicated in the process of ischemic injury. In experiments using mice, Zhang et al. (2014) observed that inhibiting the binding of HMGB1 to TLR4 downregulates IL-17A levels, thereby inhibiting neuronal apoptosis, improving nerve repair and reducing infarct volume. By blocking the TLR4 receptor, the extent of brain tissue edema, infarct size, and increased neurological damage scores after stroke are reduced *in vivo*, which possibly occurs *via* the TLR4/MyD88 signal pathway. Therefore, HMGB1 participates in the destruction of the BBB after stroke and leads to inflammation in brain tissue.

### The Role of HMGB1 in the Advanced Stages of Ischemic Stroke

During the advanced stages of ischemic stroke, the function of HMGB1 is not fully understood. At present, it is generally thought that HMGB1 can promote the regeneration of nerve cells, remodeling of blood vessels and recovery of neurological function in the late infarct stage (Le et al., 2018). Stroke-activated astrocytes increase the viability and migration of endogenous endothelial progenitor cells (EPCs) by releasing HMGB1, and promote neurovascular repair after stroke, while inhibition of HMGB1 by an siRNA restrains the EPC proliferation, blocks the peri-infarct angiogenesis, and increases neurological scores. Similarly, exogenous EPC transplantation promotes the regeneration of blood vessels in the ischemic region during the chronic phase of stroke, reduces the volume of brain atrophy, and improves neurological function, mainly through the involvement of HMGB1-RAGE initiated MAPK kinase/extracellular signal-regulated kinase (MEK/ERK) pathway (Hayakawa et al., 2012).

Moreover, another important role of HMGB1 after stroke is to promote endothelial cell sprouting. When different concentrations of HMGB1 are used to treat human umbilical vein endothelial cells, the degree of endothelial cell sprouting is directly proportional to the HMGB1 concentration. Schlueter et al. (2005) reported that high concentrations of HMGB1 have proinflammatory effects that cause endothelial damage, while low concentrations of HMGB1 improves EPCs activity, then promoting neurovascular growth. Thus, HMGB1 released from ischemic brain mediates post-stroke angiogenesis at the advanced stage, subsequently promoting brain repair and disease recovery.

### The Mechanisms of HMGB1 Participating in Ischemic Stroke

At present, although a pivotal role of HMGB1 in cerebral ischemia is widely accepted, the specific mechanisms are not yet fully understood. However, possible mechanisms will be discussed in the following paragraphs.

#### Regulation of Inflammation by HMGB1

As mentioned previously, post-ischemic inflammation appears to be a critical component of the progression of pathogenic stroke. Recent studies agree that HMGB1 is a recognized proinflammatory factor in ischemic stroke and positively correlates with stroke severity in animal models and patients (Harris et al., 2012; Le et al., 2018). HMGB1, as an endogenous inflammatory mediator, is passively released by necrotic cells or actively secreted by macrophages/monocytes into the ischemic core, triggering and amplifying inflammatory processes. In turn, the released HMGB1 also induces the activation of microglia, macrophages, and endothelial cells among others (Wang C. et al., 2016), which results in the production of proinflammatory mediators such as TNF-α, iNOS, IFN-γ, NO, chemokines, and cell adhesion molecules. On one hand, these proinflammatory mediators recruit more immune cells from the circulatory system into the CNS, thus aggravating the development of inflammation in the brain (Young et al., 2008). On the other hand, these proinflammatory mediators also stimulate microglia and macrophages to actively secrete HMGB1 in a positive feedback loop, which exerts an effect as late inflammatory mediators. Xiong et al. (2016) demonstrated that microglia/macrophages express HMGB1 within the ischemic core. However, when HMGB1 levels are reduced, infiltration of microglia/macrophages and leukocytes in ischemic brain tissue is significantly inhibited (Gu et al., 2013). Several studies have demonstrated 2 peaks of HMGB1 levels in CSF and serum post-MCAO (Abraham et al., 2000; Kim et al., 2006; Kim I. D. et al., 2018; Umahara et al., 2018), which is likely to be related to two separate sources of circulating HMGB1. The first peak of HMGB1 may originate from necrotic neurons and activated microglia/macrophages of the CNS, and the other may be derived from active secretion by delayed activated inflammatory cells in the postischemic hemisphere or peripheral immune cells (Le et al., 2018). This suggests that accumulated extracellular HMGB1 may not only mediate acute damaging processes in the brain but also aggravate inflammation in the brain and increase vulnerability to post-stroke infection (PSI) Recently, a study reported that HMGB1 can exacerbate inflammatory damage to the BBB during the process of brain ischemia–reperfusion, which may be the cause of the release of HMGB1 from the brain to the CSF and circulation (Li M. et al., 2018).

**Figure 3:**
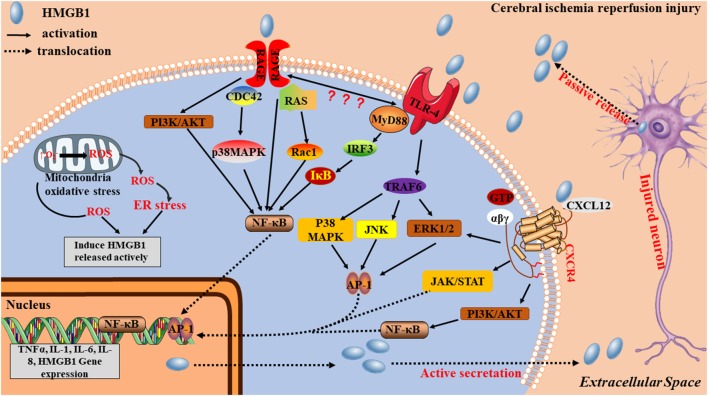
Potential mechanisms by which HMGB1 contributes to stroke pathogenesis. HMGB1 acts as an early mediator at the initial stages of stroke. An ischemia causes nerve cell injury, which leads to the passive release of the disulfide form of hypoacetylated HMGB1 from damaged cells. Extracellular HMGB1, which is either acetylated or oxidized at residues 23 and 45 to its the disulfide form, stimulates inflammatory signaling by binding to cell-surface receptors TRL4, receptor for advanced glycation end product (RAGE), and CXCR4 on microglia. Acetylated or disulfide HMGB1, together with cytokines and danger-associated molecular patterns (DAMPs), is also actively released from activated immune cells, causing additional nerve cell damage and microglia activation *via* positive feedback, thereby acting as a late inflammatory mediator.

HMGB1 is “sticky” and binds to a variety of different molecules including RACE, TLRs and CXCR4 on the cell surface. This “sticky” may partially explain the limitation of the diffusion of extracellular HMGB1, then localizing the damaging effects of HMGB1. After ischemic stroke, extracellular HMGB1 works as a DAMP, through three signaling pathways (Figure 3): (1) by binding to TLR4, HMGB1 induces MyD88 or Toll/IL-1 receptor domain-containing adaptor-inducing IFN-β (TRIF) signaling cascades, leading to the activation of transcription factors, such as NF-κB and activator protein-1 (AP-1) *via* TNF receptor-associated factor 6 (TRAF6)-mediated JNK, p38 MAPK, and ERK signaling activation (O’Neill and Bowie, 2007); (2) by interacting with RAGE, PI3K/Akt and MAPK pathways are activated, resulting in nuclear NF-κB translocation and production of inflammatory mediators including TNF-α and IL-1β (Zhang et al., 2017). PI3K/Akt pathway is usually considered to be an antiapoptotic pathway, but there is also evidence to suggest that it can facilitate the production of inflammatory cytokines (Xue et al., 2017; Li H. et al., 2018; Xu et al., 2018). Some studies have reported that when HMGB1 binds to RAGE, it also generates direct intracellular signaling resulting in nuclear NF-κB translocation (Li et al., 2016). However, other studies have suggested that TLR4 is required for HMGB1-induced NF-κB activation and cytokine formation *via* the RAGE receptor (Yang et al., 2010) and (3) when bound to CXCR4, the ERK, cyclooxygenase2/janus kinase/signal transducer and activator of transcription (COX2/JAK/STAT), and PI3K/Akt signaling pathways are activated (Schiraldi et al., 2012; Yamamoto and Tajima, 2017; Cecchinato et al., 2018), contributing to inflammatory cell migration and inflammatory mediator production.

#### Excitotoxic Injury Caused by HMGB1 Release

HMGB1 can induce excitatory neurotransmitter release in the brain after stroke. Studies have shown that HMGB1 inhibits mouse neural glial glutamate transporters by glutamate/aspartate transporter (GLAST) neural activation particles and increases extracellular levels of glutamate and its receptor. The activation of glutamate receptors causes Ca^2+^ influx, eventually leading to Ca^2+^ overload and a loss of cell function due to dyshomeostasis (Zhang J. et al., 2011). An intravenous injection of anti-HMGB1 monoclonal antibody could, therefore, reduce cerebral infarct volume and improve neurological function by preventing the elevation of glutamate levels and reducing excitotoxicity in neurons.

#### BBB Damage by HMGB1 Release

HMGB1, as an inflammatory cytokine, also can contribute to BBB breakdown, and BBB permeability is significantly reduced by using of anti-HMGB1 monoclonal antibody in experimental stroke models (Zhang J. et al., 2011). Ischemic stroke results in upregulation of MMPs, then increasing BBB permeability and even BBB damage because MMPs can break down a myriad of extracellular matrix (ECM). MMPs are usually confined to the cytosol in their inactivated state and are activated by plasmin or other MMPs. Sapojnikova et al. (2014) demonstrated that HMGB1 levels are strongly correlated with MMP-9 secretion. Qiu et al. (2010) reported that HMGB1 upregulates MMP-9 *via* the TLR4 signaling pathway; when TLR4 signaling is blocked, HMGB1-induced MMP-9 upregulation is mostly suppressed. In addition, Tissue-type plasminogen activator (tPA) is a putative pharmacotherapy for ischemic stroke, but the neurovasculature complications including edema and hemorrhagic transformation, due to BBB damage, also can occur following the using of this drug, along with the reperfusion of blood flow. When using the HMGB1-binding heptamer peptide (HBHP) to inhibit HMGB1 activity, significantly improves the BBB leakage, rescues the loss of occludin, a tight junction protein, and promotes BBB integrity, thereby reducing the complications resulted from tPA treatment (Li M. et al., 2018).

#### Regulation of Autophagy by HMGB1

The release of HMGB1 is also related to the functional state of autophagy in the early stages of stroke. There is some evidence to suggest that ischemic stroke postprocessing forms a two-way feedback mechanism to protect the brain, restraining autophagy and reducing the secretion of HMGB1. However, the relationship between HMGB1 and autophagy needs to be explored. Wang J. et al. (2016) indicated that the expression and location of HMGB1 are closely related to autophagy; remote ischemic preconditioning (RIPerC) and ischemic postconditioning (IPOC) has been shown to inhibit autophagy, blocking the translocation of HMGB1 from the nucleus to the cytoplasm. It is also clear that the inhibition of autophagy reduces the secretion of HMGB1; the reduction in HMGB1 secretion, in turn, causes the inhibition of autophagy (Hayakawa et al., 2013). Tang et al. (2010) demonstrated that the different redox states of HMGB1 lead to different cellular effects, which may be the crucial function of HMGB1: to promote information transfer between autophagy and apoptosis. Thiol dehydrogenation forms a disulfide bond (for example, when it is oxidized) between C23 and C45 of the HMGB1 protein A-box, which is required for induction of autophagy because HMGB1 binds to Beclin-1, causing Beclin-1 to separate from Bcl-2 (Tang et al., 2016).

#### Relationship Between HMGB1 and Mitochondrial Oxidative Stress/ERS

Endoplasmic reticulum (ER) and mitochondria constituted the centers of metabolic networks. After a stroke, mitochondrial oxidative stress results in a large number of ROS production, which can induce ER stress (ERS), finally causing inflammation and cell apoptosis, *via* C/EBP homologous protein (CHOP), Caspase12, JUN activation (López-Hernández et al., 2015; Poone et al., 2015). As mitochondria are quantifiable sources of ROS, and ER is a factory to folding, processing and trafficking of proteins, immune-metabolic dysregulation can occur in these compartments of neurons during cerebral ischemia–reperfusion (Narne et al., 2017; Wang et al., 2018). In response to ERS and mitochondrial oxidative stress, macrophages and monocytes actively release HMGB1. Tang et al. (2007) reported that oxidative stress plays a potential role in the regulation of HMGB1 release. They illuminated that a ROS, H_2_O_2,_ stimulates HMGB1 release actively from macrophages and monocytes, possibly *via* MAPK- and CRM1-dependent pathways (Tang et al., 2007). In addition, a recent study have shown that oxygenized low density lipoprotein (OxLDL), a risk factor of stroke, stimulates HMGB-1 secretion in macrophages resulted from oxidative stress, then HMGB1 can contribute to macrophage-derived foam cells formation *via* ERS/CHOP pathway (Wu et al., 2018).

#### Relationship Between the Immune System and HMGB1

HMGB1 an extracellular signal molecule and inflammatory mediator has been demonstrated to participate in the adaptive immune response not only by indirectly acting on dendritic cells (DCs; Yang et al., 2007) but also by directly acting on T cells to regulate their function (Dumitriu et al., 2005). The effect of HMGB1 on T cells is bidirectional (Sundberg et al., 2009). Low dose, short-term *in vitro* HMGB1 stimulation promotes the activation, maturation, and migration of DCs (Dumitriu et al., 2007). This HMGB1 stimulation can also act directly on T cells (Zhang Y. et al., 2011), stimulating T cell activation and proliferation, and polarization of (T helper cells 1) Th1, resulting in the secretion of IL-2 and INF-γ, as well as inhibiting the expression of cytotoxic T-lymphocyte-associated protein 4 (CTLA-4) and forkhead or winged helix transcription factor proteins 3 (Foxp3) in regulatory T cells and reducing the secretion of IL-10 (Wang et al., 2008; Zhu et al., 2011). In contrast, long-term, high doses of HMGB1 have the opposite effects, directly or indirectly inhibiting the activation and proliferation of DCs and Th1 polarization of T cells (Huang et al., 2009), reducing IL-2 secretion, thus blocking T cell function and inducing immunosuppression (Zhang et al., 2008; Zhu et al., 2009; Wild et al., 2012).

##### The Effect of HMGB1 on the Ischemic Brain *via* T Lymphocytes

Currently, activated T lymphocytes are considered to be a key factor in secondary tissue injury, affecting the prognosis of stroke. Xiong et al. (2016) reported that T cells participate in HMGB1 effects on the ischemic brain in both *in vivo* and *in vitro* experiments. The study showed that the HMGB1 inhibitor glycyrrhizin protects against ischemia partly by inhibiting the infiltration of T cells and their subtypes into the ischemic brain. Injection of glycyrrhizin reduces the infarct size in WT mice but not T or B cell-deficient severe combined immune deficiency (SCID) mice, while restoring T and B cells in SCID mice elicit a reduction in infarct size following glycyrrhizin treatment. The *in vitro* experiments demonstrated that glycyrrhizin inhibits neuronal death in a splenocyte and neuron coculture system with splenocytes derived from WT mice, but not from SCID mice. These results suggest a pivotal role for T cells in the detrimental effects of HMGB1 in the brain after ischemic stroke (Xiong et al., 2016).

##### Role of HMGB1 in Stroke-Induced Immunodepression

Ischemic stroke initiates not only a brain inflammation that causing cerebral injury, but also a stroke-induced immunodepression (SIID), resulting in an incidence of PSI, most typically urinary tract infections and pneumonia (Gu et al., 2015). PSI is one of the leading causes of delayed death in stroke patients (Prass et al., 2003; Emsley and Hopkins, 2010). Prass et al. (2003) first reported that the PSI is closely associated with the reduction of lymphocytes resulted from apoptosis in the peripheral organs in 2003, which is simultaneously accompanied by the shift of cytokine component from proinflammatory to anti-inflammatory profile. After that, SIID is commonly accepted, which is characterized by lymphopenia and dysfunction of lymphocytes. It is believed that HMGB1 may be related to lymphopenia and immune-function failure after ischemic stroke. Then, the correlation between levels of HMGB1 and the number of immune cells in the blood after stroke was investigated (Gu et al., 2013). When compared with the sham group, the number of total peripheral blood mononuclear cells (PBMCs) is significantly reduced while plasma HMGB1 levels increases, which suggests that HMGB1 may regulate lymphopenia and immunodepression (Gu et al., 2013). These findings are supported by a report that HMGB1 release into plasma is reduced after splenectomy, which has been previously shown to reduce infarct size and improve lymphopenia after stroke (Juneja et al., 1995; Milićević et al., 2001; Ajmo et al., 2008). Furthermore, treatment with glycyrrhizin to inhibit HMGB1 activity limits the release of HMGB1 into the blood and attenuates the reduction of total PBMCs (Gu et al., 2013; Xiong et al., 2016). All of these results confirmed the negative correlation between HMGB1 release and lymphopenia, suggesting that HMGB1 participates in the process of SIID. HMGB1 was reported to bind to RAGE or activate the TLR4/MyD88 pathway, both of which promote the reduction of mature monocytes and lymphocytes in the circulation, and lead to subsequent post-stroke immunosuppression (Huang et al., 2009; Wild et al., 2012). In addition, a recent study has shown that alarmin HMGB1 aggravates brain and systemic inflammation *via* TLR4-dependent pathway in a rat PSI model induced by lipopolysaccharides (LPS), which forms a positive feedback loop between PSI-mediated HMGB1 release and following HMGB1-release-induced enhancement of LPS function and deteriorative PSI (Kim I. D. et al., 2018).

## Conclusion and Perspective

At present, the incidence of ischemic stroke is not only increasing year by year but also becoming more severe. Ischemic stroke treatment is limited and the mortality and disability rate is extremely high. The study of ischemic stroke still requires further work. A growing body of evidence supports the idea that HMGB1 is a cytokine that regulates inflammation and immune response. It can mediate and amplify the inflammatory response after ischemia and aggravate brain damage, as well as exacerbate SIID and PSI. HMGB1 may be a potential biomarker for independently predicting the poor stroke prognosis because its upregulated blood lever is positively correlated with the infarct volume, neurological deficiency degree, BBB damage and serum inflammatory cytokine production (Le et al., 2018). However, the distribution and functions of HMGB1 are varied, but its biological mechanism is not yet clear, particularly regarding the relationship between HMGB1 and stroke. It is therefore important to study the mechanisms of HMGB1 in the absence of stroke, with the aim of using HMGB1 as a target for stroke treatments and provide new prospects and directions for stroke diagnosis and prognosis.

## Author Contributions

XX and LG designed this review article. YY wrote the manuscript. ZZ and HZ and TJ polished the article.

## Conflict of Interest Statement

The authors declare that the research was conducted in the absence of any commercial or financial relationships that could be construed as a potential conflict of interest.

## References

[B1] Abraham E. Arcaroli J. Carmody A. Wang H. Tracey K. J. (2000). HMG-1 as a mediator of acute lung inflammation. J. Immunol. 165, 2950–2954. 10.4049/jimmunol.165.6.2950 10975801

[B2] Agnello D. Wang H. Yang H. Tracey K. J. Ghezzi P. (2002). HMGB-1, a DNA-binding protein with cytokine activity, induces brain TNF and IL-6 production and mediates anorexia and taste aversion. Cytokine 18, 231–236. 10.1006/cyto.2002.0890 12126646

[B3] Ajmo C. T. Jr. Vernon D. O. Collier L. Hall A. A. Garbuzova-Davis S. Willing A.. (2008). The spleen contributes to stroke-induced neurodegeneration. J. Neurosci. Res. 86, 2227–2234. 10.1002/jnr.21661 18381759 PMC2680137

[B4] Akirav E. M. Preston-Hurlburt P. Garyu J. Henegariu O. Clynes R. Schmidt A. M.. (2012). RAGE expression in human T cells: a link between environmental factors and adaptive immune responses. PLoS One 7:e34698. 10.1371/journal.pone.0034698 22509345 PMC3324532

[B5] Andersson U. Yang H. Harris H. (2018). High-mobility group box 1 protein (HMGB1) operates as an alarmin outside as well as inside cells. Semin. Immunol. 38, 40–48. 10.1016/j.smim.2018.02.011 29530410

[B6] Ballarin B. Tymianski M. (2018). Discovery and development of NA-1 for the treatment of acute ischemic stroke. Acta Pharmacol. Sin. 39, 661–668. 10.1038/aps.2018.5 29565039 PMC5943917

[B7] Balosso S. Liu J. Bianchi M. E. Vezzani A. (2014). Disulfide-containing high mobility group box-1 promotes N-methyl-D-aspartate receptor function and excitotoxicity by activating toll-like receptor 4-dependent signaling in hippocampal neurons. Antioxid. Redox Signal. 21, 1726–1740. 10.1089/ars.2013.5349 24094148

[B8] Bao M. H. Szeto V. Yang B. B. Zhu S. Z. Sun H. S. Feng Z. P. (2018). Long non-coding RNAs in ischemic stroke. Cell Death Dis. 9:281. 10.1038/s41419-018-0282-x 29449542 PMC5833768

[B9] Bianchi M. E. Crippa M. P. Manfredi A. A. Mezzapelle R. Rovere Querini P. Venereau E. (2017). High-mobility group box 1 protein orchestrates responses to tissue damage via inflammation, innate and adaptive immunity and tissue repair. Immunol. Rev. 280, 74–82. 10.1111/imr.12601 29027228

[B10] Carty M. Bowie A. G. (2011). Evaluating the role of toll-like receptors in diseases of the central nervous system. Biochem. Pharmacol. 81, 825–837. 10.1016/j.bcp.2011.01.003 21241665

[B11] Cecchinato V. D’Agostino G. Raeli L. Nerviani A. Schiraldi M. Danelon G.. (2018). Redox-mediated mechanisms fuel monocyte responses to CXCL12/HMGB1 in active rheumatoid arthritis. Front. Immunol. 9:2118. 10.3389/fimmu.2018.02118 30283452 PMC6157448

[B12] Chen Z. Venkat P. Seyfried D. Chopp M. Yan T. Chen J. (2017). Brain-heart interaction: cardiac complications after stroke. Circ. Res. 121, 451–468. 10.1161/CIRCRESAHA.117.311170 28775014 PMC5553569

[B13] Choi J. Y. Cui Y. Chowdhury S. T. Kim B. G. (2018). High-mobility group box-1 as an autocrine trophic factor in white matter stroke. Proc. Natl. Acad. Sci. U S A 114, E4987–E4995. 10.1073/pnas.1702035114 28584116 PMC5488940

[B14] Cully M. (2013). Connective tissue diseases: HMGB1 helps elicit anti-dsDNA antibody production in SLE. Nat. Rev. Rheumatol. 9:321. 10.1038/nrrheum.2013.75 23670137

[B15] Dumitriu I. E. Baruah P. Valentinis B. Voll R. E. Herrmann M. Nawroth P. P.. (2005). Release of high mobility group box 1 by dendritic cells controls T cell activation via the receptor for advanced glycation end products. J. Immunol. 174, 7506–7515. 10.4049/jimmunol.174.12.7506 15944249

[B16] Dumitriu I. E. Bianchi M. E. Bacci M. Manfredi A. A. Rovere-Querini P. (2007). The secretion of HMGB1 is required for the migration of maturing dendritic cells. J. Leukoc. Biol. 81, 84–91. 10.1189/jlb.0306171 17035340

[B17] Emsley H. C. Hopkins S. J. (2010). Post-stroke immunodepression and infection: an emerging concept. Infect. Disord. Drug Targets 10, 91–97. 10.2174/187152610790963528 20166972

[B18] Fann D. Y. Lee S. Y. Manzanero S. Chunduri P. Sobey C. G. Arumugam T. V. (2013). Pathogenesis of acute stroke and the role of inflammasomes. Ageing Res. Rev. 12, 941–966. 10.1016/j.arr.2013.09.004 24103368

[B19] Faraco G. Fossati S. Bianchi M. E. Patrone M. Pedrazzi M. Sparatore B.. (2007). High mobility group box 1 protein is released by neural cells upon different stresses and worsens ischemic neurodegeneration *in vitro* and *in vivo*. J. Neurochem. 103, 590–603. 10.1111/j.1471-4159.2007.04788.x 17666052

[B20] Gardella S. Andrei C. Ferrera D. Lotti L. V. Torrisi M. R. Bianchi M. E.. (2002). The nuclear protein HMGB1 is secreted by monocytes via a non-classical, vesicle-mediated secretory pathway. EMBO Rep. 3, 995–1001. 10.1093/embo-reports/kvf198 12231511 PMC1307617

[B21] Goldstein R. S. Gallowitsch-Puerta M. Yang L. Rosas-Ballina M. Huston J. M. Czura C. J.. (2006). Elevated high-mobility group box 1 levels in patients with cerebral and myocardial ischemia. Shock 25, 571–574. 10.1097/01.shk.0000209540.99176.72 16721263

[B22] Goodwin G. H. Sanders C. Johns E. W. (1973). A new group of chromatin-associated proteins with a high content of acidic and basic amino acids. Eur. J. Biochem. 38, 14–19. 10.1111/j.1432-1033.1973.tb03026.x 4774120

[B23] Gu L. Jian Z. Stary C. Xiong X. (2015). T cells and cerebral ischemic stroke. Neurochem. Res. 40, 1786–1791. 10.1007/s11064-015-1676-0 26220139

[B24] Gu L. Xiong X. Wei D. Gao X. Krams S. Zhao H. (2013). T cells contribute to stroke-induced lymphopenia in rats. PLoS One 8:e59602. 10.1371/journal.pone.0059602 23555048 PMC3598760

[B25] Harris H. E. Andersson U. Pisetsky D. S. (2012). HMGB1: a multifunctional alarmin driving autoimmune and inflammatory disease. Nat. Rev. Rheumatol. 8, 195–202. 10.1038/nrrheum.2011.222 22293756

[B26] Hayakawa K. Pham L. D. Arai K. Lo E. H. (2013). High-mobility group box 1: an amplifier of stem and progenitor cell activity after stroke. Acta Neurochir. Suppl. 118, 31–38. 10.1007/978-3-7091-1434-6_5 23564100 PMC3985720

[B27] Hayakawa K. Pham L. D. Katusic Z. S. Arai K. Lo E. H. (2012). Astrocytic high-mobility group box 1 promotes endothelial progenitor cell-mediated neurovascular remodeling during stroke recovery. Proc. Natl. Acad. Sci. U S A 109, 7505–7510. 10.1073/pnas.1121146109 22529378 PMC3358881

[B28] Hori O. Brett J. Slattery T. Cao R. Zhang J. Chen J. X.. (1995). The receptor for advanced glycation end products (RAGE) is a cellular binding site for amphoterin. Mediation of neurite outgrowth and co-expression of rage and amphoterin in the developing nervous system. J. Biol. Chem. 270, 25752–25761. 10.1074/jbc.270.43.25752 7592757

[B29] Hossain D. M. S. Javaid S. Cai M. Zhang C. Sawant A. Hinton M.. (2018). Dinaciclib induces immunogenic cell death and enhances anti-PD1-mediated tumor suppression. J. Clin. Invest. 128, 644–654. 10.1172/JCI94586 29337311 PMC5785250

[B30] Hua F. Ma J. Ha T. Xia Y. Kelley J. Williams D. L.. (2007). Activation of toll-like receptor 4 signaling contributes to hippocampal neuronal death following global cerebral ischemia/reperfusion. J. Neuroimmunol. 190, 101–111. 10.1016/j.jneuroim.2007.08.014 17884182 PMC2453597

[B31] Huang L. F. Yao Y. M. Zhang L. T. Dong N. Yu Y. Sheng Z. Y. (2009). The effect of high-mobility group box 1 protein on activity of regulatory T cells after thermal injury in rats. Shock 31, 322–329. 10.1097/SHK.0b013e3181834070 18665051

[B32] Jian Z. Ding S. Deng H. Wang J. Yi W. Wang L.. (2016). Probenecid protects against oxygen-glucose deprivation injury in primary astrocytes by regulating inflammasome activity. Brain Res. 1643, 123–129. 10.1016/j.brainres.2016.05.002 27154322

[B33] Juneja S. Januszewicz E. Wolf M. Cooper I. (1995). Post-splenectomy lymphocytosis. Clin. Lab. Haematol. 17, 335–337. 8697729

[B34] Kang J. W. Lee S. M. (2012). Melatonin inhibits type 1 interferon signaling of toll-like receptor 4 via heme oxygenase-1 induction in hepatic ischemia/reperfusion. J. Pineal Res. 53, 67–76. 10.1111/j.1600-079x.2012.00972.x 22288937

[B39] Kim S. W. Jin Y. Shin J. H. Kim I. D. Lee H. K. Park S.. (2012). Glycyrrhizic acid affords robust neuroprotection in the postischemic brain via anti-inflammatory effect by inhibiting HMGB1 phosphorylation and secretion. Neurobiol. Dis. 46, 147–156. 10.1016/j.nbd.2011.12.056 22266336

[B36] Kim I. D. Lee H. Kim S. W. Lee H. K. Choi J. Han P. L.. (2018). Alarmin HMGB1 induces systemic and brain inflammatory exacerbation in post-stroke infection rat model. Cell Death Dis. 9:426. 10.1038/s41419-018-0438-8 29555931 PMC5859283

[B37] Kim J. B. Lim C. M. Yu Y. M. Lee J. K. (2008). Induction and subcellular localization of high-mobility group box-1 (HMGB1) in the postischemic rat brain. J. Neurosci. Res. 86, 1125–1131. 10.1002/jnr.21555 17975839

[B35] Kim E. J. Park S. Y. Baek S. E. Jang M. A. Lee W. S. Bae S. S.. (2018). HMGB1 increases IL-1β production in vascular smooth muscle cells via nlrp3 inflammasome. Front. Physiol. 9:313. 10.3389/fphys.2018.00313 29643819 PMC5882820

[B38] Kim J. B. Sig Choi J. Yu Y. M. Nam K. Piao C. S. Kim S. W.. (2006). HMGB1, a novel cytokine-like mediator linking acute neuronal death and delayed neuroinflammation in the postischemic brain. J. Neurosci. 26, 6413–6421. 10.1523/JNEUROSCI.3815-05.2006 16775128 PMC6674036

[B40] Kokkola R. Andersson A. Mullins G. Ostberg T. Treutiger C. J. Arnold B.. (2005). RAGE is the major receptor for the proinflammatory activity of HMGB1 in rodent macrophages. Scand. J. Immunol. 61, 1–9. 10.1111/j.0300-9475.2005.01534.x 15644117

[B41] Laird M. D. Shields J. S. Sukumari-Ramesh S. Kimbler D. E. Fessler R. D. Shakir B.. (2014). High mobility group box protein-1 promotes cerebral edema after traumatic brain injury via activation of toll-like receptor 4. Glia 62, 26–38. 10.1002/glia.22581 24166800 PMC4503251

[B42] Le K. Mo S. Lu X. Idriss Ali A. Yu D. Guo Y. (2018). Association of circulating blood HMGB1 levels with ischemic stroke: a systematic review and meta-analysis. Neurol. Res. 40, 907–916. 10.1080/01616412.2018.1497254 30015578

[B44] Li M. Chen S. Shi X. Lyu C. Zhang Y. Tan M.. (2018). Cell permeable HMGB1-binding heptamer peptide ameliorates neurovascular complications associated with thrombolytic therapy in rats with transient ischemic stroke. J. Neuroinflammation 15:237. 10.1186/s12974-018-1267-5 30139371 PMC6108117

[B45] Li X. Hu X. Wang J. Xu W. Yi C. Ma R.. (2016). Short-term hesperidin pretreatment attenuates rat myocardial ischemia/reperfusion injury by inhibiting high mobility group box 1 protein expression via the PI3K/Akt pathway. Cell. Physiol. Biochem. 39, 1850–1862. 10.1159/000447884 27744432

[B43] Li H. Xie S. Qi Y. Li H. Zhang R. Lian Y. (2018). TNF-α increases the expression of inflammatory factors in synovial fibroblasts by inhibiting the PI3K/AKT pathway in a rat model of monosodium iodoacetate-induced osteoarthritis. Exp. Ther. Med. 16, 4737–4744. 10.3892/etm.2018.6770 30542428 PMC6257214

[B46] Liesz A. Dalpke A. Mracsko E. Antoine D. J. Roth S. Zhou W.. (2015). DAMP signaling is a key pathway inducing immune modulation after brain injury. J. Neurosci. 35, 583–598. 10.1523/JNEUROSCI.2439-14.2015 25589753 PMC4293412

[B47] Liu K. Mori S. Takahashi H. K. Tomono Y. Wake H. Kanke T.. (2007). Anti-high mobility group box 1 monoclonal antibody ameliorates brain infarction induced by transient ischemia in rats. FASEB J. 21, 3904–3916. 10.1096/fj.07-8770com 17628015

[B48] Liu Y. Prasad R. Wilson S. H. (2010). HMGB1: roles in base excision repair and related function. Biochim. Biophys. Acta 1799, 119–130. 10.1016/j.bbagrm.2009.11.008 20123074 PMC2818529

[B49] Lo E. H. (2010). Degeneration and repair in central nervous system disease. Nat. Med. 16, 1205–1209. 10.1038/nm.2226 21052074 PMC3985732

[B50] Lok K. Z. Basta M. Manzanero S. Arumugam T. V. (2015). Intravenous immunoglobulin (IVIg) dampens neuronal toll-like receptor-mediated responses in ischemia. J. Neuroinflammation 12:73. 10.1186/s12974-015-0294-8 25886362 PMC4409750

[B51] López-Hernández B. Ceña V. Posadas I. (2015). The endoplasmic reticulum stress and the HIF-1 signalling pathways are involved in the neuronal damage caused by chemical hypoxia. Br. J. Pharmacol. 172, 2838–2851. 10.1111/bph.13095 25625917 PMC4439879

[B52] Lorenzen I. Mullen L. Bekeschus S. Hanschmann E. M. (2017). Redox regulation of inflammatory processes is enzymatically controlled. Oxid. Med. Cell. Longev. 2017:8459402. 10.1155/2017/8459402 29118897 PMC5651112

[B53] Lotze M. T. Tracey K. J. (2005). High-mobility group box 1 protein (HMGB1): nuclear weapon in the immune arsenal. Nat. Rev. Immunol. 5, 331–342. 10.1038/nri1594 15803152

[B54] Majumdar M. Ratho R. Chawla Y. Singh M. P. (2013). High levels of circulating HMGB1 as a biomarker of acute liver failure in patients with viral hepatitis E. Liver Int. 33, 1341–1348. 10.1111/liv.12197 23682703

[B55] Milićević N. M. Luettig B. Trautwein C. Wustefeld T. Mähler M. Jecker P.. (2001). Splenectomy of rats selectively reduces lymphocyte function-associated antigen 1 and intercellular adhesion molecule 1 expression on B-cell subsets in blood and lymph nodes. Blood 98, 3035–3041. 10.1182/blood.v98.10.3035 11698288

[B56] Muhammad S. Barakat W. Stoyanov S. Murikinati S. Yang H. Tracey K. J.. (2008). The HMGB1 receptor RAGE mediates ischemic brain damage. J. Neurosci. 28, 12023–12031. 10.1523/JNEUROSCI.2435-08.2008 19005067 PMC4597312

[B57] Narne P. Pandey V. Phanithi P. B. (2017). Interplay between mitochondrial metabolism and oxidative stress in ischemic stroke: an epigenetic connection. Mol. Cell. Neurosci. 82, 176–194. 10.1016/j.mcn.2017.05.008 28552342

[B58] Neumann S. Shields N. J. Balle T. Chebib M. Clarkson A. N. (2015). Innate immunity and inflammation post-stroke: an α7-nicotinic agonist perspective. Int. J. Mol. Sci. 16, 29029–29046. 10.3390/ijms161226141 26690125 PMC4691088

[B59] Okuma Y. Liu K. Wake H. Zhang J. Maruo T. Date I.. (2012). Anti-high mobility group box-1 antibody therapy for traumatic brain injury. Ann. Neurol. 72, 373–384. 10.1002/ana.23602 22915134

[B60] O’Neill L. A. Bowie A. G. (2007). The family of five: TIR-domain-containing adaptors in toll-like receptor signalling. Nat. Rev. Immunol. 7, 353–364. 10.1038/nri2079 17457343

[B61] Park J. S. Svetkauskaite D. He Q. Kim J. Y. Strassheim D. Ishizaka A.. (2004). Involvement of toll-like receptors 2 and 4 in cellular activation by high mobility group box 1 protein. J. Biol. Chem. 279, 7370–7377. 10.1074/jbc.M306793200 14660645

[B62] Poone G. K. Hasseldam H. Munkholm N. Rasmussen R. S. Gronberg N. V. Johansen F. F. (2015). The hypothermic influence on CHOP and Ero1-α in an endoplasmic reticulum stress model of cerebral ischemia. Brain Sci. 5, 178–187. 10.3390/brainsci5020178 25989620 PMC4493463

[B63] Prass K. Meisel C. Höflich C. Braun J. Halle E. Wolf T.. (2003). Stroke-induced immunodeficiency promotes spontaneous bacterial infections and is mediated by sympathetic activation reversal by poststroke T helper cell type 1-like immunostimulation. J. Exp. Med. 198, 725–736. 10.1084/jem.20021098 12939340 PMC2194193

[B64] Qin Y. H. Dai S. M. Tang G. S. Zhang J. Ren D. Wang Z. W.. (2009). HMGB1 enhances the proinflammatory activity of lipopolysaccharide by promoting the phosphorylation of MAPK p38 through receptor for advanced glycation end products. J. Immunol. 183, 6244–6250. 10.4049/jimmunol.0900390 19890065

[B65] Qiu J. Nishimura M. Wang Y. Sims J. R. Qiu S. Savitz S. I.. (2008). Early release of HMGB-1 from neurons after the onset of brain ischemia. J. Cereb. Blood Flow Metab. 28, 927–938. 10.1038/sj.jcbfm.9600582 18000511

[B66] Qiu J. Xu J. Zheng Y. Wei Y. Zhu X. Lo E. H.. (2010). High-mobility group box 1 promotes metalloproteinase-9 upregulation through toll-like receptor 4 after cerebral ischemia. Stroke 41, 2077–2082. 10.1161/STROKEAHA.110.590463 20671243 PMC3066477

[B67] Rauvala H. Rouhiainen A. (2007). RAGE as a receptor of HMGB1 (Amphoterin): roles in health and disease. Curr. Mol. Med. 7, 725–734. 10.2174/156652407783220750 18331230

[B68] Sapojnikova N. Kartvelishvili T. Asatiani N. Zinkevich V. Kalandadze I. Gugutsidze D.. (2014). Correlation between MMP-9 and extracellular cytokine HMGB1 in prediction of human ischemic stroke outcome. Biochim. Biophys. Acta 1842, 1379–1384. 10.1016/j.bbadis.2014.04.031 24815357

[B69] Scaffidi P. Misteli T. Bianchi M. E. (2002). Release of chromatin protein HMGB1 by necrotic cells triggers inflammation. Nature 418, 191–195. 10.1038/nature00858 12110890

[B70] Schiraldi M. Raucci A. Muñoz L. M. Livoti E. Celona B. Venereau E.. (2012). HMGB1 promotes recruitment of inflammatory cells to damaged tissues by forming a complex with CXCL12 and signaling via CXCR4. J. Exp. Med. 209, 551–563. 10.1084/jem.20111739 22370717 PMC3302219

[B71] Schlueter C. Weber H. Meyer B. Rogalla P. Roser K. Hauke S.. (2005). Angiogenetic signaling through hypoxia: HMGB1: an angiogenetic switch molecule. Am. J. Pathol. 166, 1259–1263. 10.1016/s0002-9440(10)62344-9 15793304 PMC1602384

[B72] Schulze J. Zierath D. Tanzi P. Cain K. Shibata D. Dressel A.. (2013). Severe stroke induces long-lasting alterations of high-mobility group box 1. Stroke 44, 246–248. 10.1161/STROKEAHA.112.676072 23204053 PMC3530419

[B73] Seidu R. A. Wu M. Su Z. Xu H. (2017). Paradoxical role of high mobility group box 1 in glioma: a suppressor or a promoter? Oncol. Rev. 11:325. 10.4081/oncol.2017.325 28382190 PMC5364998

[B74] Shichita T. Ito M. Morita R. Komai K. Noguchi Y. Ooboshi H.. (2017). MAFB prevents excess inflammation after ischemic stroke by accelerating clearance of damage signals through MSR1. Nat. Med. 23, 723–732. 10.1038/nm.4312 28394332

[B75] Shichita T. Sakaguchi R. Suzuki M. Yoshimura A. (2012). Post-ischemic inflammation in the brain. Front. Immunol. 3:132. 10.3389/fimmu.2012.00132 22833743 PMC3400935

[B76] Singh V. Roth S. Veltkamp R. Liesz A. (2016). HMGB1 as a key mediator of immune mechanisms in ischemic stroke. Antioxid. Redox Signal. 24, 635–651. 10.1089/ars.2015.6397 26493086

[B77] Sparatore B. Patrone M. Passalacqua M. Pedrazzi M. Gaggero D. Pontremoli S.. (2001). Extracellular processing of amphoterin generates a peptide active on erythroleukaemia cell differentiation. Biochem. J. 357, 569–574. 10.1042/bj3570569 11439110 PMC1221987

[B78] Stonesifer C. Corey S. Ghanekar S. Diamandis Z. Acosta S. A. Borlongan C. V. (2017). Stem cell therapy for abrogating stroke-induced neuroinflammation and relevant secondary cell death mechanisms. Prog. Neurobiol. 158, 94–131. 10.1016/j.pneurobio.2017.07.004 28743464 PMC5671910

[B79] Stros M. (2010). HMGB proteins: interactions with DNA and chromatin. Biochim. Biophys. Acta 1799, 101–113. 10.1016/j.bbagrm.2009.09.008 20123072

[B80] Sundberg E. Fasth A. E. Palmblad K. Harris H. E. Andersson U. (2009). High mobility group box chromosomal protein 1 acts as a proliferation signal for activated T lymphocytes. Immunobiology 214, 303–309. 10.1016/j.imbio.2008.09.006 19201506

[B81] Tang D. Kang R. Livesey K. M. Cheh C. W. Farkas A. Loughran P.. (2010). Endogenous HMGB1 regulates autophagy. J. Cell Biol. 190, 881–892. 10.1083/jcb.200911078 20819940 PMC2935581

[B82] Tang D. Shi Y. Kang R. Li T. Xiao W. Wang H.. (2007). Hydrogen peroxide stimulates macrophages and monocytes to actively release HMGB1. J. Leukoc. Biol. 81, 741–747. 10.1189/jlb.0806540 17135572 PMC1808495

[B83] Tang Y. Zhao X. Antoine D. Xiao X. Wang H. Andersson U.. (2016). Regulation of posttranslational modifications of HMGB1 during immune responses. Antioxid. Redox Signal. 24, 620–634. 10.1089/ars.2015.6409 26715031 PMC5349223

[B84] Tao X. Sun X. Yin L. Han X. Xu L. Qi Y.. (2015). Dioscin ameliorates cerebral ischemia/reperfusion injury through the downregulation of TLR4 signaling via HMGB-1 inhibition. Free Radic. Biol. Med. 84, 103–115. 10.1016/j.freeradbiomed.2015.03.003 25772012

[B85] Thomas J. O. Stott K. (2012). H1 and HMGB1: modulators of chromatin structure. Biochem. Soc. Trans. 40, 341–346. 10.1042/BST20120014 22435809

[B86] Tirone M. Tran N. L. Ceriotti C. Gorzanelli A. Canepari M. Bottinelli R.. (2018). High mobility group box 1 orchestrates tissue regeneration via CXCR4. J. Exp. Med. 215, 303–318. 10.1084/jem.20160217 29203538 PMC5748844

[B87] Tong S. Zhang L. Joseph J. Jiang X. (2018). Celastrol pretreatment attenuates rat myocardial ischemia/reperfusion injury by inhibiting high mobility group box 1 protein expression via the PI3K/Akt pathway. Biochem. Biophys. Res. Commun. 497, 843–849. 10.1016/j.bbrc.2018.02.121 29475002

[B88] Tsoyi K. Nizamutdinova I. T. Jang H. J. Mun L. Kim H. J. Seo H. G.. (2010). Carbon monoxide from CORM-2 reduces HMGB1 release through regulation of IFN-β/JAK2/STAT-1/INOS/NO signaling but not COX-2 in TLR-activated macrophages. Shock 34, 608–614. 10.1097/SHK.0b013e3181e46f15 20442692

[B89] Tsung A. Klune J. R. Zhang X. Jeyabalan G. Cao Z. Peng X.. (2007). HMGB1 release induced by liver ischemia involves toll-like receptor 4 dependent reactive oxygen species production and calcium-mediated signaling. J. Exp. Med. 204, 2913–2923. 10.1084/jem.20070247 17984303 PMC2118528

[B90] Umahara T. Uchihara T. Hirokawa K. Hirao K. Shimizu S. Hashimoto T.. (2018). Time-dependent and lesion-dependent HMGB1-selective localization in brains of patients with cerebrovascular diseases. Histol. Histopathol. 33, 215–222. 10.14670/HH-11-914 28671243

[B91] Venereau E. Casalgrandi M. Schiraldi M. Antoine D. J. Cattaneo A. De Marchis F.. (2012). Mutually exclusive redox forms of HMGB1 promote cell recruitment or proinflammatory cytokine release. J. Exp. Med. 209, 1519–1528. 10.1084/jem.20120189 22869893 PMC3428943

[B94] Wang J. Han D. Sun M. Feng J. (2016). A combination of remote ischemic perconditioning and cerebral ischemic postconditioning inhibits autophagy to attenuate plasma HMGB1 and induce neuroprotection against stroke in rat. J. Mol. Neurosci. 58, 424–431. 10.1007/s12031-016-0724-9 26852332

[B92] Wang C. Jiang J. Zhang X. Song L. Sun K. Xu R. (2016). Inhibiting HMGB1 reduces cerebral ischemia reperfusion injury in diabetic mice. Inflammation 39, 1862–1870. 10.1007/s10753-016-0418-z 27596007 PMC5112296

[B93] Wang C. S. Meng D. Yang H. Wang X. Jia S. Wang Y.-F. (2018). Pathological basis of cardiac arrhythmias: vicious cycle of immune-metabolic dysregulation. Cardiovasc. Dis. Med. 3, 1–7. 10.15761/CDM.1000158

[B95] Wang Z. T. Yao Y. M. Sheng Z. Y. (2008). Effect of high mobility group box 1 protein on proliferation and apoptosis and balance between Th1/Th2 and Tc1/Tc2 of lymphocytes *in vitro*. Xi Bao Yu Fen Zi Mian Yi Xue Za Zhi 24, 324–327. 10.13423/j.cnki.cjcmi.004642 18394333

[B96] Wild C. A. Bergmann C. Fritz G. Schuler P. Hoffmann T. K. Lotfi R.. (2012). HMGB1 conveys immunosuppressive characteristics on regulatory and conventional T cells. Int. Immunol. 24, 485–494. 10.1093/intimm/dxs051 22473704

[B97] Wu H. Chen Z. Chen J. Z. Pei L. G. Xie J. Wei Z. H.. (2018). High mobility group B-1 (HMGB-1) promotes apoptosis of macrophage-derived foam cells by inducing endoplasmic reticulum stress. Cell. Physiol. Biochem. 48, 1019–1029. 10.1159/000491970 30041247

[B99] Xiong X. X. Gu L. J. Shen J. Kang X. H. Zheng Y. Y. Yue S. B.. (2014). Probenecid protects against transient focal cerebral ischemic injury by inhibiting HMGB1 release and attenuating AQP4 expression in mice. Neurochem. Res. 39, 216–224. 10.1007/s11064-013-1212-z 24317635

[B98] Xiong X. Gu L. Wang Y. Luo Y. Zhang H. Lee J.. (2016). Glycyrrhizin protects against focal cerebral ischemia via inhibition of T cell activity and HMGB1-mediated mechanisms. J. Neuroinflammation 13:241. 10.1186/s12974-016-0705-5 27609334 PMC5016958

[B100] Xu T. Qin G. Jiang W. Zhao Y. Xu Y. Lv X. (2018). 6-Gingerol protects heart by suppressing myocardial ischemia/reperfusion induced inflammation via the PI3K/Akt-dependent mechanism in rats. Evid. Based Complement. Alternat. Med. 2018:6209679. 10.1155/2018/6209679 30519268 PMC6241357

[B101] Xue J. F. Shi Z. M. Zou J. Li X. L. (2017). Inhibition of PI3K/AKT/mTOR signaling pathway promotes autophagy of articular chondrocytes and attenuates inflammatory response in rats with osteoarthritis. Biomed. Pharmacother. 89, 1252–1261. 10.1016/j.biopha.2017.01.130 28320092

[B102] Yamamoto T. Tajima Y. (2017). HMGB1 is a promising therapeutic target for acute liver failure. Expert Rev. Gastroenterol. Hepatol. 11, 673–682. 10.1080/17474124.2017.1345625 28657371

[B104] Yang H. Antoine D. J. Andersson U. Tracey K. J. (2013). The many faces of HMGB1: molecular structure-functional activity in inflammation, apoptosis and chemotaxis. J. Leukoc. Biol. 93, 865–873. 10.1189/jlb.1212662 23446148 PMC4051189

[B103] Yang D. Chen Q. Yang H. Tracey K. J. Bustin M. Oppenheim J. J. (2007). High mobility group box-1 protein induces the migration and activation of human dendritic cells and acts as an alarmin. J. Leukoc. Biol. 81, 59–66. 10.1189/jlb.0306180 16966386

[B105] Yang H. Hreggvidsdottir H. S. Palmblad K. Wang H. Ochani M. Li J.. (2010). A critical cysteine is required for HMGB1 binding to toll-like receptor 4 and activation of macrophage cytokine release. Proc. Natl. Acad. Sci. U S A 107, 11942–11947. 10.1073/pnas.1003893107 20547845 PMC2900689

[B108] Yang Q. W. Lu F. L. Zhou Y. Wang L. Zhong Q. Lin S.. (2011). HMBG1 mediates ischemia-reperfusion injury by TRIF-adaptor independent toll-like receptor 4 signaling. J. Cereb. Blood Flow Metab. 31, 593–605. 10.1038/jcbfm.2010.129 20700129 PMC3049514

[B106] Yang H. Lundback P. Ottosson L. Erlandsson-Harris H. Venereau E. Bianchi M. E.. (2012). Redox modification of cysteine residues regulates the cytokine activity of high mobility group box-1 (HMGB1). Mol. Med. 18, 250–259. 10.2119/molmed.2011.00389 22105604 PMC3324950

[B107] Yang H. Wang H. Ju Z. Ragab A. A. Lundback P. Long W.. (2015). MD-2 is required for disulfide HMGB1-dependent TLR4 signaling. J. Exp. Med. 212, 5–14. 10.1084/jem.20141318 25559892 PMC4291531

[B109] Young V. G. Halliday G. M. Kril J. J. (2008). Neuropathologic correlates of white matter hyperintensities. Neurology 71, 804–811. 10.1212/01.wnl.0000319691.50117.54 18685136

[B111] Zhang J. Takahashi H. K. Liu K. Wake H. Liu R. Maruo T.. (2011). Anti-high mobility group box-1 monoclonal antibody protects the blood-brain barrier from ischemia-induced disruption in rats. Stroke 42, 1420–1428. 10.1161/STROKEAHA.110.598334 21474801

[B112] Zhang J. Wu Y. Weng Z. Zhou T. Feng T. Lin Y. (2014). Glycyrrhizin protects brain against ischemia-reperfusion injury in mice through HMGB1-TLR4-IL-17A signaling pathway. Brain Res. 1582, 176–186. 10.1016/j.brainres.2014.07.002 25111887

[B110] Zhang B. Yang N. Mo Z. M. Lin S. P. Zhang F. (2017). IL-17A enhances microglial response to OGD by regulating p53 and PI3K/Akt pathways with involvement of ROS/HMGB1. Front. Mol. Neurosci. 10:271. 10.3389/fnmol.2017.00271 28912678 PMC5583146

[B113] Zhang L. T. Yao Y. M. Dong Y. Q. Dong N. Yu Y. Sheng Z. Y. (2008). Relationship between high-mobility group box 1 protein release and T-cell suppression in rats after thermal injury. Shock 30, 449–455. 10.1097/SHK.0b013e3181672495 18277947

[B114] Zhang Y. Yao Y. M. Huang L. F. Dong N. Yu Y. Sheng Z. Y. (2011). The potential effect and mechanism of high-mobility group box 1 protein on regulatory T cell-mediated immunosuppression. J. Interferon Cytokine Res. 31, 249–257. 10.1089/jir.2010.0019 21087077

[B115] Zhu X. M. Yao Y. M. Liang H. P. Xu C. T. Dong N. Yu Y.. (2011). High mobility group box-1 protein regulate immunosuppression of regulatory T cells through toll-like receptor 4. Cytokine 54, 296–304. 10.1016/j.cyto.2011.02.017 21419643

[B116] Zhu X. M. Yao Y. M. Liang H. P. Xu S. Dong N. Yu Y.. (2009). The effect of high mobility group box-1 protein on splenic dendritic cell maturation in rats. J. Interferon Cytokine Res. 29, 677–686. 10.1089/jir.2008.0104 19642897

